# Exploring the potential of ChatGPT for foreign language education at the university level

**DOI:** 10.3389/fpsyg.2024.1269319

**Published:** 2024-04-08

**Authors:** Blanka Klimova, Marcel Pikhart, Liqaa Habeb Al-Obaydi

**Affiliations:** ^1^Department of Applied Linguistics, University of Hradec Kralove Rokitanskeho, Hradec Kralove, Czechia; ^2^English Department, College of Education for Human Sciences, University of Diyala, Baqubah, Iraq

**Keywords:** AI technology, ChatGPT, university students, foreign language learning, L2 acquisition

## Abstract

**Introduction:**

The purpose of this study is to explore students’ attitudes and perceived usefulness of using ChatGPT for learning a foreign language to reveal how this new trend tool affects its end-users.

**Methods:**

The authors conducted qualitative research by using a questionnaire survey based on hands-on experience by university students.

**Results:**

The findings reveal that students are fascinated, satisfied, and stimulated to use this technology despite some of their reservations and potential threats. The authors of this study also list pedagogical implications, including specific activities, while using ChatGPT.

**Discussion:**

Although ChatGPT can be very beneficial for teachers and help them in their preparation, it sets a challenging task for them to change the existing teaching approaches and assessments to boost students’ cognitive, creative, and critical thinking skills. In addition, both teachers and students will have to upskill their competencies to handle the current advancements in AI technology, such as ChatGPT.

## 1 Introduction

Artificial intelligence (AI) started to seriously affect people’s lives. Its use undoubtedly has an enormous impact on almost every area of modern society (Naidu, 2019), including foreign language education. AI tools, such as neural machine translation or chatbots, enable more personalized and self-regulated learning if students are properly guided by their teacher. For example, chatbots may improve students’ language skills, especially their speaking as research studies indicate (Gayed et al., 2022). In addition, they can provide automated corrective feedback, which can stimulate students to learn a foreign language (Smutny and Schreiberova, 2020; Klimova and Pikhart, 2022). Thus, it can reduce teachers’ burden of grading hundreds of papers (Reiss, 2021). On the contrary, however, the use of AI tools can pose certain risks and threats, such as misuse of personal and private data or incorrect information (Klimova et al., 2023).

This is especially true for the very recent AI tool, the so-called ChatGPT, which was introduced in November 2022 and immediately caused a stir. ChatGPT is a large language model developed by OpenAI. Dowling and Lucey (2023) reviewed this tool and came to the conclusion that ChatGPT can generate reasonable research ideas, literature reviews, and test suggestions, but to produce a quality research study, it would need expert knowledge and a larger dataset. In addition, the use of ChatGPT for research purposes raises several ethical questions, such Is it proper to have such an advanced level of guidance and assistance, and still claim the produced research as one’s own? Could ChatGPT help to flatten the disparities between the global south and wealthier nations in terms of research output?

In foreign language education teachers will have to re-evaluate teaching approaches and in particular, assessment methods since the use of ChatGPT enables students to easily generate logically structured and rigorous professional-based essays (IPL, 2023). However, on the contrary, it is a beneficial tool for students to quickly acquire reliable responses to their concept-based general questions, as well as help them to enhance their writing skills if facilitated efficiently by their teachers (Kasneci et al., 2023). Firat (2023) listed some of the potentials of ChatGPT for educational purposes, such as personalized learning since each student can have different needs and Chat GPT can easily react to this; real-time feedback on the task performance; convenient and flexible learning because students can use it at any time and anywhere; enhancing the use of open educational resources or self-reflection on one’s own progress.

The concerns described above, coupled with the potential of ChatGPT to disrupt traditional assessment methods and enhance learning outcomes, necessitate a study into how such tools are perceived and utilized by learners. The proposed qualitative study aims to fill this gap by examining students’ attitudes toward ChatGPT and its perceived use in learning a foreign language. Understanding these perceptions is vital for developing effective teaching strategies that leverage AI’s benefits while mitigating its risks. This research could also contribute to the broader discourse on AI in education, offering insights into how these technologies can be integrated ethically and effectively to support diverse learning needs and goals.

The rationale of the paper lies in the increasing integration of artificial intelligence, particularly tools like ChatGPT, into foreign language education. It acknowledges the potential benefits of AI in language learning, such as personalized learning, real-time feedback, and flexibility in accessing educational resources. However, it also acknowledges the risks associated with AI tools, including ethical concerns and potential disruptions to traditional assessment methods. The paper contributes to foreign language learning by addressing the gap in understanding students’ attitudes towards and perceptions of ChatGPT in language education. By exploring these attitudes and perceptions, the study aims to inform the development of effective teaching strategies that harness the benefits of AI while addressing its limitations and ethical considerations. The proposed qualitative study seeks to answer specific research questions aimed at understanding students’ attitudes toward ChatGPT, its benefits and limitations in language learning, and the pedagogical implications for teaching in the era of AI. By addressing these questions, the study aims to provide insights into how AI technologies like ChatGPT can be ethically and effectively integrated into language education to support diverse learning needs and goals. Thus, the paper’s rationale lies in exploring the potential of ChatGPT in foreign language education, addressing both its benefits and limitations, and providing insights into how teachers can effectively utilize AI technologies to enhance language learning outcomes while navigating ethical considerations.

To verify the aims of the study, the following research questions were set:

1. How do students perceive the integration of ChatGPT in foreign language learning contexts?
2. How does ChatGPT enhance foreign language acquisition for students?
3. What challenges do students encounter when utilizing ChatGPT for learning a foreign language?
4. How can the presence of ChatGPT influence pedagogical approaches in teaching foreign languages?

## 2 Literature review

### 2.1 Use of chatbots in foreign language education

Chatbots are among the most important emerging developments in language learning, or at least they may be. Chatbots are an illustration of a macro-and micro-level AI (Artificial Intelligence) application utilized in the classroom or even outside to assist students in developing their speaking, reading, writing, and listening skills, among other language-related talents (Gayed et al., 2022). Using text and/or speech, a chatbot can simulate human-like, natural conversations with people, according to Ashfaque et al. (2020). This is where human-computer interaction comes into play. The chat system may be text-based or task-based, and it may respond with speech, graphics, virtual motions, or even physically by making tactual gestures (Belda-Medina and Calvo-Ferrer, 2022).

Interaction with teachers, peers, and other professionals is essential for language learning. Interaction is crucial for the process of learning a language because it gives students the ability to provide updated output, receive feedback on their output, and intelligible input (Liu, 2022). Interacting with conversational or pedagogical chatbots, especially AI chatbots, can offer learners these opportunities for language acquisition (Mageira et al., 2022). Because they provide immediate and realistic communication with students in their target language and, intelligent chatbots are piquing the interest of language teachers worldwide (Lee et al., 2020; Voinohovska and Doncheva, 2022). According to several research studies, chatbots may enhance pupils’ language abilities, particularly their speaking (Gayed et al., 2022). To this end, old and new chatbot applications are distinguished from one another. Early chatbots were built on a set of pre-established principles that were derived from outside information. They were therefore not very “intelligent” and unable to react to questions that did not fall under their predetermined scope of operation. Contrarily, the current implementation of artificial intelligence (AI-driven chatbots) supports giving rich output, which is essential for the success of second language learning. As a result, they may have intelligent conversations with people, continuously pick up new information from past interactions, grow over time, and operate as devoted language learning assistants (Fryer et al., 2019). According to Huang et al. (2022), these complex chatbots may provide advanced linguistic input and output, imitate daily conversation practice, and pique language learners’ attention. Chatbots can help users become more motivated and engaged, two factors that are essential for technology-supported language acquisition (Petrović and Jovanović, 2021).

The relevant literature (Klimova and Pikhart, 2022; Klimova et al., 2023) highlights the positive aspects of AI tools, such as chatbots and neural machine translation in enhancing the language learning experience. These tools can provide personalized learning, and automated corrective feedback and thus reduce the burden on teachers (Kim, 2019; Grassini, 2023). These tools are potentially utilizable in an educational context but further research is required to evaluate the benefits for students and to evaluate how could AI positively contribute to language learning.

### 2.2 Use of ChatGPT in specific foreign language teaching scenarios

ChatGPT is a well-known and very sophisticated chatbot that supports AI. It is one of the most advanced AI-powered chatbots now which was developed by Microsoft-backed startup OpenAI, called ChatGPT (generative pre-trained transformer), and it was released in November 2022. Although chatbots have been used in educational settings since the early 1970s (Huang et al., 2022), ChatGPT’s ability to faithfully mimic human interaction opens up a novel and exciting path for language learning. By accurately simulating real-world interactions, ChatGPT promotes language learning. It can interpret a word’s meaning in light of its context, clarify grammatical mistakes, write texts of various genres (including emails, stories, and recipes), make quizzes, annotate texts, and offer dictionary definitions, model sentences, and translations (Kohnke et al., 2023). Even though all of these advantages and more are stated by studies, many of them also draw attention to their drawbacks and risks, such as plagiarism (Kohnke et al., 2023), cheating, answer accuracy, cultural prejudice (Rettberg, 2022), and the subjective satisfaction of students with online education (Pikhart et al., 2022), all these drawbacks encourage closer attention to the use of it in educational settings and pave the way for additional research to better understand the situation and ensure the utmost caution is used when dealing with it.

One of the core benefits of using ChatGPT in foreign language teaching scenarios is its ability to simulate conversational interactions. Learners can engage in dialogues with the AI, practicing their speaking and comprehension skills in a safe, judgment-free environment. This is crucial for building fluency and confidence, as students can experiment with vocabulary, grammar, and sentence structures without the fear of making mistakes in front of peers (Barrot, 2023). Furthermore, ChatGPT’s capacity to generate diverse scenarios and conversations allows learners to encounter a wide range of linguistic contexts, enhancing their ability to comprehend and communicate in a foreign language under different circumstances (Dwivedi et al., 2023).

For instance, Xiao and Zhi (2023) in their qualitative research study point out that their learners appreciated using ChatGPT since it operates as an educational companion or individual instructor by delivering tailored, conveniently obtainable, and adaptable responses. Additionally, it aids in enhancing linguistic aptitude when learners employ analytical reasoning abilities, for example, adjusting prompts, instructing the system, and assessing and judiciously approving its results. Lastly, it enables the generation of ideas for brainstorming. In the same vein, maintain that students have the opportunity to work at their own pace and focus on the specific areas where they require the greatest assistance, ultimately leading to a learning environment that is both more impactful and efficient. On the other hand, the findings of Algaraady and Mahyoob (2023) indicate that although ChatGPT may serve as a beneficial instrument in discerning superficial mistakes, it is incapable of substituting the proficiency and intricate comprehension possessed by human educators when it comes to recognizing errors associated with the more intricate elements of written expression.

Thus, while chatbots like ChatGPT represent a significant advancement in AI and language learning, their integration into educational settings must be approached with caution. The benefits of enhanced interactivity, adaptability, and engagement must be weighed against the risks of limited scope in early models, plagiarism, cheating, accuracy issues, and cultural biases (*cf.* Grassini, 2023). This balance is essential to harness the full potential of chatbots in language learning while mitigating their drawbacks. Future research and careful implementation strategies are necessary to ensure that chatbots are used effectively and responsibly in educational contexts (Kooli, 2023).

### 2.3 Methodology

The research sample consisted of 91 undergraduate students of Management of Tourism and Economics and Management at the Faculty of Informatics and Management at the University of Hradec Kralove, Czech Republic. All these students study English as an applied language and their level of English ranges between B2 and C1. All respondents provided their informed consent and the research was approved by the Ethics Committee of the University of Hradec Kralove no. 4/2023. No personal or private data were collected and the GDPR standards were followed.

This study is based on a qualitative research design to examine new areas of inquiry, i.e., the potential of ChatGPT for foreign language learning (FLE). This approach allowed the authors of this study to explore complex issues of the potential of ChatGPT for FLE in depth, offering a nuanced understanding of this AI tool from the perspective of the study’s participants. Out of the qualitative methods that include interviews, focus groups, qualitative surveys, participant observations, or content analysis, the authors used the qualitative survey with open-ended questions to collect a wide range of responses on this research topic, as well as to identify themes, patterns, and insights into participants’ experiences.

In February 2023, students were asked to fill in an online questionnaire survey about their use of ChatGPT. However, before its implementation, the content of the questionnaire was consulted with other experienced colleagues from the department. In addition, 10 students tried out the piloted version to eliminate potential shortcomings and ensure its reliability and validity. The questionnaire included 12 open-ended questions, which were as follows:

1. When did you first hear about ChatGPT and what was your first experience when you used it?
2. Please describe your first feelings and evaluation of using ChatGPT.
3. How satisfied were you with the performance of ChatGPT when you used it?
4. Be specific about what tasks it performed well and what did not too much.
5. What do/did you find useful about it?
6. How could you use it in your life, now or in the future? For what specific activities?
7. What do you find strange or challenging when using ChatGPT?
8. Are there any specific situations when you did not feel comfortable when using ChatGPT?
9. Do you generally like ChatGPT? If so, why? If not, why not?
10. Do you miss any specific functionality in ChatGPT you would like to have there?
11. Is there any worry about ChatGPT you have?
12. How can it help you and other students when studying foreign languages and other subjects?

Prior to the questionnaire, the students were given these five tasks to complete using ChatGPT as a tool to solve these issues:

- Ask ChatGPT to explain to you how to use zero, definite, and indefinite articles in English.
- Ask ChatGPT to write a short article (about 500) on the topic of your choice and provide references.
- Ask ChatGPT to explain or describe a topic or issue, or provide an answer to the question that interests you.
- Use ChatGPT to write a complaint email to a hotel about an issue you had there. Provide the details.
- Use ChatGPT to translate any short paragraph (100–200 words) you find on the internet into Czech and check what it looks like to you. Is it natural? Grammatically correct?

After the participants tried ChatGPT, they were asked to answer the questions of the questionnaire, whose answers were based on the tasks described above and carried out in learning English as a foreign language.

## 3 Results

The results overview follows the structure of the questionnaire. The first question of the questionnaire was as follows:

*When did you first hear about ChatGPT and what was your first experience when you used it?*

Generally, many students heard about ChatGPT when it was first launched since many of them are students of ICT and therefore they are well-informed about the current trends. However, most of the students did not hear about ChatGPT until the beginning of the year 2023. Some of the participants had already experimented with the tool providing them with a useful and creative tool for their everyday activities related to their studies. They already experimented with ChatGPT when translating texts and asking it questions in another language than English, in this case, Czech. Those who tried it claimed that they had been surprised that it can communicate in Czech. Czech students are used to the fact that all the available AI-driven software, such as DeepL and Google Translate, is not very good at Czech and the mistakes are plentiful and annoying. ChatGPT, unlike, was praised for its excellent mastery of the Czech language, and the participants were fascinated that it can be used even in minority languages. Many of the participants expressed their interest in the topic and they had already searched for more information about ChatGPT, which seems to be an extremely interesting topic for them. They also reported they would use it in the future and it seems from their responses that they were fascinated more than ever before with other technological achievements.

The second question of the questionnaire was related to the subjective evaluation of the tool after experimenting with the tool fulfilling the assignments and the question was as follows:

*Please describe your first feelings and evaluation of using ChatGPT.*

First impression counts and the first reactions of all participants who encountered this technology were extremely positive, some of them stating how impressed they were by its flawless execution of the tasks and also its response time. But there were also mixed feelings expressed that this is going to change the world in a good and bad way at the same time. Among the issues reported, there were only a few ideas mentioned, such as incompatibility with a particular browser or the fact that all available slots were used and it was not possible to use the app. Some respondents also realized that there would be a serious problem with cheating while using ChatGPT. Moreover, they also realized that it would not be good for the enhancement of their cognitive abilities because the ChatGPT would do the task instead of them. They mentioned several industries where the tool could easily be implemented, such as education, healthcare, science, and ICT. One of the participants summarizes it clearly stating “*I think it can be a very helpful tool, however, I am a bit wary of it, since it can lead to people being lazy and using it to create articles and reports, or possibly even academic papers, which scares me. It surprised me with how quickly it can come up with answers to complex questions and I’m still not sure how I feel about that.*”

The third question of the questionnaire focused on the subjective satisfaction of the users with ChatGPT when performing the given tasks.

*How satisfied were you with the performance of ChatGPT when you used it?*

Again, the users were very or even extremely satisfied with the tool comparing it to previously used tools, such as Google search or user forums where the information was obtained but not as conveniently as from ChatGPT. The only issue reported was limited response time due to its busyness. Regarding its answers in Czech, there were several issues with respect to language accuracy but as it is a minority language the results are still impressive. Some participants have noted that ChatGPT can explain their questions but in a somehow limited way, as a computer – they confirmed that a teacher can explain in a better way providing more viewpoints and possible explanations so that everyone can understand. The computer, on the other hand, tends to repeat the same thing even if it asks you to rephrase it as you do not understand. One of the weaknesses that were spotted was the fact that the interface of ChatGPT is – for now – only in written format, it was noted that if there were other outputs, such as audio or visual, it would bring it to another level. All in all, the general satisfaction was on an extremely high level due to its accuracy, and speed, but also the human-like nature of the way information was provided as stated by one of the respondents: “*I was very surprised about the level Chat GPT can reply to my questions with perfect English but I still believe that the teachers will be needed in the future because they provide more human approach.”*

The following question 4 aimed at providing specific details related to the performance of ChatGPT as it is evaluated by the users who use it for the first time and they can compare the tasks and their level of completion as it is evaluated by their subjective level of satisfaction.

*Be specific about what tasks it performed well and what tasks did not do too much.*

Again, the evaluation of ChatGPT was generally very positive stating that it can provide any information that is available on the internet, particularly topics related to Science, Maths, or Computer science. However, for writing an academic text, ChatGPT was not considered ideal as it does not provide correct and current references. Moreover, it only provided basic information that is easily accessible on Wikipedia and similar websites. It was also highly appreciated to be used for writing professional emails and technical reports with the specialized technical language although the user does not have sufficient expertise for this text. Explaining a topic and writing a paragraph or an article were rated very high. However, some participants admitted that it would be much better for them to have the explanation from a teacher, or at least a YouTube video teacher who could do a much better job. Some students also admitted that they would use ChatGPT to help them with their Bachelor’s thesis and they have already tried it with very good results. It also can provide references to the cited sources. The biggest problem reported repeatedly is a translation that does not make sense lexically and grammatically. It was graded on the same level as Google Translate and also had problems with minority languages. The translated result was not natural, full of mistakes, and difficult to understand in some cases, but others reported only minor language issues related to the unnatural tone of the translation (“the language sounds too computerized and unnatural”). However, grammar correction was rated again on a high level (“I cannot see any grammar of vocabulary problems, it seems perfect, much better than my own language performance – I am astonished”).

Some of the students wanted to try ChatGPT as a tool for writing creative stories and in this task, it performed excellently. The users stated that the story was very natural and human-like not being able to see that it was machine-created. In addition, for sophisticated discussion on a political or philosophical topic, it was rated highly positive (“it is so smart when I ask any questions, even complicated and wise”). The format in bullet points and the structured way were also appreciated as it did not provide lengthy answers but readily accessible structured information.

Question five was related to usefulness as it was perceived by the users.

*What do/did you find useful about it?*

The respondents rated the usefulness on a very high level because it can make work more efficient and faster in many ways, it can produce very good quality information in a very short time, and in many ways, it can save people’s work as well. The most useful application will be explaining, writing, copyediting, and other text-processing-related areas. It can save a lot of time when searching for information that is available online or that needs some analysis.

For a programmer, it can also be a very useful tool that can spot mistakes in a code or even generate a code on its own based on the request which is can save a significant amount of time in some professions (“I am shocked how it is possible that it can create a code in such a speed and it is perfect”). When looking for specific information, it is much faster than Google as it is not necessary to evaluate the information google provides on many websites but ChatGPT collects all the information and digests it for the user providing the sources so that the user can see where the information was extracted from.

Some participants also mentioned that ChatGPT can be used for inspiration, such as providing help with simple life questions like what to cook for lunch, or what to buy for a young brother for a birthday (“I can even ask it to solve simple issues of my daily life, such as cooking tips and shopping ideas”). In this task, ChatGPT performed also very well providing reasonable and creative ideas based on the information provided. For some of them, ChatGPT can even be something like a friend to talk to, but this opinion was not very common.

Surprisingly, rarely anyone mentions that there is a very thin line between cheating and moral integrity when using ChatGPT and everyone only stated that it is very useful for creating presentations, writing seminar papers, and Bachelor theses without asking a question whether this use is not beyond ethical standards and plagiarism. Stating that “*I can save time for more important things*” does not seem justifiable and sufficient. As many of the users mention that it can easily be used for writing essays, none of them has asked a question whether this behavioral is ethical or even beneficial for student development.

The following question of the questionnaire dealt with the practical utilization of ChatGPT as it is perceived by the students.

*How could you use it in your life, now or in the future? For what specific activities?*

As many of the respondents are ICT students, they already use it and will use it for help in the coding to spot issues in their code. Similarly, the majority of them already use ChatGPT to search for information and they claim that they will also use it to write emails, which could save them time. They also want to use it to write their essays so that they do not need to do it themselves. The translation is also mentioned as an area that will be covered by ChatGPT. Many of the users claim that it allows them to save time by using ChatGPT because the tasks are done immediately and without mistakes or just minor ones, such as writing essays, emails, translating, and searching for information (“The outcome is perfect, I will save so much time now when I can use it for translations and other tasks I previously had to do myself”).

Not many respondents realized that they could use it for their improvement and education. Those who stated they would use it for their education will use it for clarifying more complicated issues that are difficult to understand without a teacher. The most common area where ChatGPT would be used is programming, either to spot mistakes in the code or when coding is needed and it requires too much time.

Some of the users also want to use it to get more information when traveling, what places to visit, where to eat, what to buy as a souvenir, what book to read, or what films to watch. It can even help with starting a business when looking for a niche in the market. Generally, many users claim that when creating texts, can be very useful because the user can gain confidence in the form of grammatical correctness and completeness in writing different word forms. Further, students could work with this technology in the form of information retrieval for their presentations and term papers.

The most important aspect noted by the users is that it can save time compared to traditional search engines or books or even consulting a human. Looking for references that would support an argument seems also very important as Google and other search engines are not very good at it and the references they provide are not relevant and useful. Using ChatGPT as a personal assistant in planning daily activities is also mentioned. It could help with grocery shopping, finding recipes, and planning family trips. Using it for entertainment and fund activities was not mentioned many times and the users rather considered serious text editing, copyediting, and programming. The condition mentioned many times to use ChatGPT actively is the condition that it will be for free and there will not be any extra conditions when users want to use it.

*What do you find strange or challenging when using ChatGPT?*

The answers provided by ChatGPT were often considered too fast and solid, concise and consistent, and finally infallible. Some respondents realized that there was no possibility to verify the information and the threat could be the reliability of obtained information. Many reported that it felt strange to communicate with a robot and that it could finally replace people’s intelligence or it will lead to reduced intelligence in the future, people will not write essays and articles by themselves anymore, which will lead to reduced skills and abilities.

It was also noted that the strangest thing was that the discussion felt almost human as if there was someone behind the screen enabling human-like small talk with the chat. The users were often worried about their private data as there was no possibility to block the ChatGPT from storing the private data provided by the users. ChatGPT provides a lot of information without any referencing, which was considered inadequate and not appropriate. Some users reported a serious issue related to the fact that, for an enthusiast impressed by it, could be quite challenging to stop giving it more and more questions which could make it even an addiction. But the system reminds the user it is only an AI language model that cannot feel any emotions. Therefore, there is no need to worry about someone falling in love with it while chatting about different topics. Oftentimes, it was reported that it was scary that the AI one day would be able to rule humans, even though ChatGPT mentions that it is a conspiracy (“When I was using it I was always worried about what to ask. I had a feeling that someone else is watching me”).

The next question was related to the previous one and it was its extension to collect more information related to the idea of discomfort.

*Are there any specific situations when you did not feel comfortable when using ChatGPT?*

The majority of responses stated that there were no issues and related feelings of discomfort. Only a few reported that they are generally uncomfortable when using AI-driven technology as they do not agree with its implementation in the everyday lives of ordinary people. The users generally did not like the idea that they had to register with their email, full name, and phone number. The users were worried about data privacy as they were not provided with any information as to what would happen to their private data and where it is stored.

The fact that it knows everything made the users uncomfortable and they were worried that someone might track them through it and, for example, steal their social media accounts. To avoid possible discomfort, the users did not talk about sensitive or inappropriate situations noting also that the chat behaves extremely politically correct.

The following question focused on the users’ general satisfaction.

*Do you generally like ChatGPT? If so, why? If not, why not?*

Again, the users reported that they were amazed but they realized that it could be used in a dishonest way, such as cheating. They reported that they are fascinated and that it was a huge step for the future with a big impact on our lives soon but it will have a negative impact too as it could be a big threat to universities and the qualification papers of their students. The users almost always considered it a great tool that could help people in many different areas and do tasks for them and thus people can focus on more meaningful things. Those who did not share this over-optimistic opinion claimed that they were not fans of this AI boom and that they were afraid that in time civilization would lose its cleverness because an AI would do everything for them. Consequently, students will use this for all their works, essays, maybe theses, and degrees and education will lose its authority.

Subjectively evaluated usefulness reaches high levels as the users are aware that it certainly has a lot to offer to a large number of users working/studying in various fields. On the other hand, information needs to be verified and taken with a grain of salt. There are certainly many things and features that need to be worked on in the future to make this technology work even better and more accurately. However, this usefulness will necessarily create an overreliance on it. Therefore, some of them believe that it should be used as a support, rather than the main creator. The potential addiction to ChatGPT is likened to the addiction to social media and the users must be aware of it one must not forget it is still only AI and therefore the answers cannot be trusted unconditionally.

To sum it up, users like ChatGPT very much, they are even excited about it, but they also realize the potential risks that are related to its use, such as addiction, data privacy, and other threats to society.

The next question was focused on the possible functionalities that could be missing in ChatGPT.

*Do you miss any specific functionality in ChatGPT you would like to have there?*

In this question, the respondents were not very creative. As they admitted their amazement with the tool, they were not able to provide any specific functionality except for additional languages, such as Czech and other minority languages. Only a few reported that it should be able to share photos and not just plain text or generally an image function was missed there. The citation function was also missed quite frequently.

The following question is a repeated item to double-check what the worries of the users could be.

*Is there any worry about ChatGPT you have?*

Some of the users worry that society will put too much emphasis on machines which would lead to loss of job positions and that relying on technology might crumble in on itself somewhere in the future and that there is the problem of winner takes all in the tech space and the AI “war” between tech titans has just begun. Many respondents also worry that it could be harmful to teachers who check the student’s works since there is no possibility to recognize whether it is plagiarism or not. And people (students) will take advantage of this app as shortly it will be so easy to write a Bachelor’s paper, which is unfair. The major worry was, however, data privacy and being tracked by unknown corporations. Furthermore, they are worried that this technology can become more intelligent than humans and it can lead to the destruction of humankind. Another item frequently mentioned was that it can give us false information - because the data is collected from databases that are on the internet. Not all information can be considered true and some people could take it as a good source of information or they could rely on it while making a life decision. Moreover, it can also cause people in the future could lose the ability to look for information themselves. ChatGPT will soon be monetized, which can also lead to serious problems.

Students are aware that it will have a serious impact on education systems around the world as teachers will no longer be able to assign essay writing, for example, as homework for students because it would be almost impossible to detect if the text was written by its author.

Some of the most negative evaluations were related to the spread of false information stating: “*Definitely a big threat. For example, it can help spread misinformation. It certainly also expresses a great threat to education itself, which will have to change from the ground up. So-called soft skills should be given more priority. Essentially oral examination, practical education, dealing with people, working in groups and the like.”*

The final question was related to the use of ChatGPT when studying foreign languages or some other subjects at school.

*How can it help you and other students when studying foreign languages and other subjects?*

Everyone agreed that ChatGPT had brilliant explaining capabilities, however, the main advantage comes from endless conversations and/or infinite grammar examples. It can help explain the grammar or the meaning of individual words in English. As a translation tool, it is not believed it will reach the human capacity to create a professional translation. As far as other subjects are concerned, ChatGPT can be used to search for definitions or necessary references, for example, when preparing seminar papers or presentations.

As several students put it: *As long as the information exists somewhere on the internet that this AI can track it down. Be it math, statistics, or foreign language studies. What’s also useful is that it explains concepts in a short and easy-to-understand manner for the most part. It can also be a great tool for improving writing skills in English. It is possible to chat with ChatGPT and also ask for grammar corrections. It can help when learning vocabulary that is used in* var*ious situations and between different people based on their relationships. It is also good for explaining grammar and other linguistic features*. *With one click, they can have an essay or a translated text and the like. However, the ability to think critically, search for information and the like is lost. This may result in an overall decline in intelligence and a decline in the importance of attending college.*

The descriptive analysis of the results is as follows. It provides several insights into the participants’ experiences, perceptions, and attitudes toward ChatGPT. The results clearly reveal that many students, particularly those in the ICT area, had prior awareness of OpenAI projects like DALL-E. However, the majority only heard about ChatGPT a few weeks before the research. Those who experimented with the tool were pleasantly surprised by its proficiency in Czech, unlike other AI-driven software commonly used in the region. First impressions of ChatGPT were overwhelmingly positive, with users praising its flawless execution and swift response times. Despite the excitement, some participants expressed mixed feelings about the tool’s potential to impact the world both positively and negatively. Concerns included issues like incompatibility with specific browsers and potential misuse leading to laziness and academic dishonesty. Despite minor issues, user satisfaction with ChatGPT’s performance was high, especially when compared to other tools like Google Search. Users acknowledged its limitations, such as language accuracy in complex explanations and the absence of audio or visual outputs in the interface. When asked about specific tasks, ChatGPT received praise for translating texts, answering questions, and generating creative stories. However, weaknesses were identified in academic text writing, citation accuracy, and translation quality. Some users raised concerns about the tool’s potential impact on critical thinking skills and the potential for misuse in academic settings. Participants found ChatGPT highly useful for its efficiency and time-saving capabilities, particularly in professional and educational tasks. It was seen as a valuable tool for coding assistance, generating code, and providing quick answers. Users also noted its potential application in daily life decisions, such as cooking tips and shopping ideas. Ethical considerations were somewhat downplayed, with users acknowledging the potential for cheating but not explicitly discussing the ethical standards of using ChatGPT for academic tasks. Concerns were raised about the tool’s potential addiction, data privacy, and the reliability of the information provided. Looking to the future, many students, especially those in the ICT area, expressed their intentions to continue using ChatGPT for coding, searching for information, and writing essays. Despite acknowledging potential risks, users generally liked ChatGPT, expressing excitement about its capabilities while also recognizing the need for caution due to possible negative consequences, including job loss, education impact, and the spread of false information.

## 4 Discussion

As seen above, the findings generated enough answers to the research questions set in the Introduction and they are summarized and discussed in their order below.

The findings of this qualitative study indicate that students feel positive and satisfied with the use of ChatGPT since it brings many benefits to university students in enhancing their study performance, such as writing seminar papers, Bachelor theses, or simply, explaining concepts in a very fast pace This is in line with other research studies on this topic, such as Tlili et al., (2023) or Zhai (2022). For example, Zhai (2022) states that especially writing with the help of ChatGPT is extremely efficient since within a short time, the user possessing very limited professional knowledge can generate a very informative and systematic piece of professional writing. In addition, ChatGPT can help programmers detect errors in coding, as well as write these codes (*cf.* Glen, 2022). Similarly, as in the use of other very sophisticated chatbots, students find this AI tool almost human-like as far as communication is concerned (*cf.* Firat, 2023). As they stated above, they feel fascinated by the sophisticated output it can generate.

The results also reveal that the key benefits for students learning a foreign language include general information retrieval, very fast and quite easy grammar explanation, and correction, even in the native language, or essay writing as with Klimova and Al-Obaydi (2023).

With the help of the answers obtained or articles written, students can learn new vocabulary or word order in a sentence. Furthermore, students can make translations with the help of ChatGPT even in minority languages (Kasneci et al., 2023), such as Czech, and write professional e-mails and technical reports easily. It can look up articles connected to a topic that the student is passionate about, which can make studying more enjoyable, effective, and thus stimulating, which can again have a very positive impact on student achievement results (*cf.* Ofgang, 2022; Poláková and Klímová, 2022).

As far as the limitations are concerned, there is still an issue with false referencing and a lack of visual images that are so important for the visual and tactile learning styles of students. In addition, ChatGPT is not now able to analyze and evaluate empirical data and explain very specific information, as well as provide spoken interaction (*cf.* IPL, 2023). Moreover, there is a threat of the misuse and security of personal data (*cf.* Klimova et al., 2023; Tlili et al., 2023). On the other hand, students did not admit to being unethical when using ChatGPT for writing, for example, their seminar papers, but they are aware of the fact that plagiarism cannot be detected (*cf.* King and ChatGPT, 2023). For instance, Ofgang (2022) states that it is sufficient for students to type in a few quick prompts and they get the whole essay in 1–2 min without being penalized by teachers who are not able to decipher this plagiarism. In addition, since ChatGPT is very popular nowadays, its platform is frequently too occupied that it prevents its use. On the other hand, the problem of engagement in online applications is still active (Al-Obaydi et al., 2023).

All the information provided above set a very challenging task for teachers, not only those of foreign languages, but all other teacher practitioners, to rethink their teaching practices and develop more creative tasks for students to make them critically reflect on their work and stimulate them to study in a different way (Doncheva, 2014). This can be done in various ways and the authors of this study list some below:

- To set problem-solving activities,
- Develop new assessment methods, such as oral exams during which students could perform their acquired knowledge, be it through ChatGPT, in a creative way and discuss the set issue with his/her peer,
- Ask them to visualize and draw the procedure of solving the task,
- In foreign language classes reflect on written tasks done by ChatGPT and ask students how to improve the outcome of such generated tasks, or
- Simply use a flipped classroom approach, such as recording the lecture on Youtube, making them find more information via ChatGPT and in-class debate on their acquired knowledge, and make them critically evaluate it.

The research findings generated several practical implications related to the field of applied linguistics and second language acquisition as follows: The findings of this research have several practical implications for applied linguistics. First and foremost, ChatGPT’s proficiency in multiple languages, including minority languages like Czech, offers significant support for language learners. Students can utilize ChatGPT for language practice, translation tasks, and clarifying grammar and vocabulary concepts, thus augmenting their language learning experience. This aspect of the tool provides an avenue for students to engage with the target language in a meaningful way, even in contexts where resources may be limited.

Moreover, ChatGPT’s ability to generate coherent and structured texts can assist language learners in improving their writing skills. By providing immediate feedback and suggestions, ChatGPT can help students refine their writing style, grammar, and vocabulary usage, thereby enhancing their overall proficiency in the target language. This feature is particularly valuable for students who may struggle with writing tasks or who require additional support in developing their writing abilities. Additionally, while ChatGPT primarily operates in written format, its conversational capabilities can still provide opportunities for speaking practice. Students can engage in dialog with ChatGPT to practice pronunciation, fluency, and conversational skills, albeit in a simulated environment. This aspect of the tool can complement traditional speaking practice activities and provide students with additional opportunities to interact in the target language.

Furthermore, ChatGPT’s capacity to generate explanations, stories, and summaries can facilitate comprehension and content creation in language learning contexts. Learners can use ChatGPT to explain complex concepts, summarize texts, or generate creative content, thereby deepening their understanding of the language and its nuances. This feature enables students to engage with authentic language input and output, thereby promoting language acquisition and proficiency development. Moreover, the use of ChatGPT in language assessment can offer insights into students’ linguistic proficiency and writing abilities. By analyzing students’ interactions with ChatGPT and the quality of output generated, educators can assess language skills, identify areas for improvement, and tailor instruction accordingly. This aspect of the tool can streamline the assessment process and provide valuable data for informing instructional decisions.

However, the research also emphasizes the importance of addressing ethical considerations associated with the use of AI tools like ChatGPT in language education. Educators and policymakers must establish guidelines and protocols to ensure responsible and ethical use of AI technologies, particularly regarding issues of plagiarism, data privacy, and academic integrity. This aspect of the research highlights the need for ongoing dialog and collaboration among stakeholders to ensure that the benefits of AI in language education are maximized while mitigating potential risks. In summary, the findings of this research highlight the potential of ChatGPT as a valuable tool for supporting language learning and teaching in diverse contexts. However, they also underscore the need for careful consideration of ethical implications and pedagogical adaptation to ensure its responsible and effective integration into language education practices.

## 5 Conclusion

The up-to-date research on the very recent conversational chatbot – ChatGPT – shows that this AI technology revolutionized the existing educational system, as well as highlighted the perception of both its benefits and threats. Although ChatGPT can be very beneficial for teachers and help them in their preparation of teaching materials, presentations, or quizzes, it sets a challenging task for them to change the existing teaching approaches and assessments to boost students’ cognitive, creative, and critical thinking skills. Furthermore, Tlili et al. (2023) and Tawafak et al. (2023) point out, that both teachers and students will have to upskill their competencies to handle the current advancements of online education and AI technology, such as ChatGPT.

This study has some limitations. Firstly, it is a qualitative study, which means that it does not include any quantitative data to provide a more objective evaluation. Secondly, the research sample was quite homogeneous, including only university students from one country. But on the other hand, there are no studies available that would deal with the topic and this fresh reflection of the student’s feelings brings more light on the topic and will stimulate more research in this very novel area. Therefore, future research should include a more varied range of respondents, as well as focus on experimental studies to verify whether ChatGPT can offer added value to foreign language learning. Despite these shortcomings, the findings of this study form a solid base for further exploration of this very recent tool and suggest specific activities that can be immediately implemented in classes.

Future research in applied linguistics concerning ChatGPT and similar AI technologies could explore several areas. Longitudinal studies could track students’ interactions with ChatGPT over time to understand its long-term effects on language learning. Comparative studies could compare ChatGPT with traditional methods, informing instructional practices. Investigating pedagogical strategies for integrating ChatGPT into language teaching and learning contexts could enhance language learning experiences. Ethical considerations, such as plagiarism and data privacy, need further exploration to develop responsible AI use guidelines. Understanding user experience and performance across different languages would inform ChatGPT’s potential applications. Additionally, research on teacher training and support and ChatGPT’s use in language assessment could improve implementation strategies and evaluation practices. By addressing these areas, scholars can advance our understanding of AI integration in language education, leading to evidence-based practices and policies. And this research could be a foundation upon which further studies could be built.

## Data availability statement

The raw data supporting the conclusions of this article will be made available by the authors, without undue reservation.

## Ethics statement

The studies involving humans were approved by The Ethical Committee of the University of Hradec Kralove. The studies were conducted in accordance with the local legislation and institutional requirements. The participants provided their written informed consent to participate in this study.

## Author contributions

BK: Conceptualization, Data curation, Formal analysis, Investigation, Methodology, Resources, Supervision, Validation, Visualization, Writing – original draft, Writing – review & editing. MP: Data curation, Formal analysis, Investigation, Methodology, Resources, Validation, Writing – original draft, Writing – review & editing. LA-O: Formal analysis, Investigation, Resources, Writing – original draft, Writing – review & editing.
